# *α*-Synuclein ubiquitination – functions in proteostasis and development of Lewy bodies

**DOI:** 10.3389/fnmol.2024.1498459

**Published:** 2024-11-12

**Authors:** Hung-Hsiang Ho, Simon S. Wing

**Affiliations:** ^1^Department of Medicine, McGill University and Research Institute of the McGill University Health Centre, Montreal, QC, Canada; ^2^Integrated Program in Neuroscience, McGill University, Montreal, QC, Canada

**Keywords:** α-synuclein, Lewy body, ubiquitin, Parkinson’s disease, endosome, lysosome, autophagy, proteasome

## Abstract

Synucleinopathies are neurodegenerative disorders characterized by the accumulation of *α*-synuclein containing Lewy bodies. Ubiquitination, a key post-translational modification, has been recognized as a pivotal regulator of *α*-synuclein’s cellular dynamics, influencing its degradation, aggregation, and associated neurotoxicity. This review examines comprehensively the current understanding of *α*-synuclein ubiquitination and its role in the pathogenesis of synucleinopathies, particularly in the context of Parkinson’s disease. We explore the molecular mechanisms responsible for *α*-synuclein ubiquitination, with a focus on the roles of E3 ligases and deubiquitinases implicated in the degradation process which occurs primarily through the endosomal lysosomal pathway. The review further discusses how the dysregulation of these mechanisms contributes to *α*-synuclein aggregation and LB formation and offers suggestions for future investigations into the role of *α*-synuclein ubiquitination. Understanding these processes may shed light on potential therapeutic avenues that can modulate *α*-synuclein ubiquitination to alleviate its pathological impact in synucleinopathies.

## 1 Introduction

*Α*-synuclein (αS) is a presynaptic neuronal protein whose aggregation into insoluble fibrils in Lewy bodies (LBs) is a pathological hallmark of Parkinson’s disease (PD). Ubiquitination of αS was observed many years ago and subsequent research has provided evidence that this ubiquitination plays important roles in regulating αS’s stability, aggregation, and toxicity. Understanding the specific ubiquitination events that lead to αS degradation or accumulation could therefore provide valuable insights into potential therapeutic strategies to mitigate PD progression.

In this review, we provide brief overviews of ubiquitination and the role of αS in neurodegenerative diseases. We then trace the discovery of ubiquitin in LBs and its pivotal functions in proteolytic degradation pathways. The review then focuses on key studies that identify the E3 ligases (Table 1) and deubiquitinases enzymes (DUBs; Table 2) involved in αS ubiquitination and the structural and biological consequences of this ubiquitination. Finally, we discuss some knowledge gaps in this area and propose a model that integrates the role of αS ubiquitination with other contributing mechanisms in the formation of LBs (Lashuel, 2020; Moors and Milovanovic, 2024).

**Table 1:** Ubiquitin ligases for α-Syn.

Ubiquitin ligase name	Ubiquitination site on α-Syn	Research model used for Ub sites identification	Ub linkage type	Effects on αS
NEDD4/NEDD4-1	K12, K21, K45, K58, K60, K96	HEK293 cell; primary mice neuron and patient samples for K45/58/60 only	K63-polyUb chain	Promotes uptake of αS into endosomes for lysosomal degradation
SIAH-1 and SIAH-2	K10, K12, K21, K23, K34, K43, K96	Cell-free assay	MonoUb	Promotes formation of αS insoluble aggregates and/or proteasomal degradation of αS.
SCF-FBXL5	K45, K58, K60	SH-SY5Y cell	K48-K63 branched chain	Promotes degradation of αS fibrils through both proteasomal and lysosomal degradation pathways.
LUBAC	M1	SH-SY5Y cell, primary mice neuron, patient samples	Linear polyUb chain	Ubiquitinates αS aggregates for p62-dependent autophagic/lysosomal degradation (aggrephagy).
CHIP	Unknown		Monoubiquitination	Inhibits αS oligomerization potentially through association with HSC70 and promotes proteasomal/lysosomal degradation.
Parkin	Unknown		PolyUb; Linkage type unknown	Specifically targets O-linked glycosylated αS for ubiquitination, but the consequence is unknown.
E6-AP	Unknown		Linkage type unknown	Decreases endogenous αS olgiomerization likely by enhancing proteasomal degradation.

**Table 2:** Deubiquitinases for α-Syn.

Deubiquitinase Name	Effects on αS
USP9X	Stabilizes αS by possibly preventing its degradation. Degradation pathway remains unknown.
UCH-L1 (PGP9.5)	Increases aS insolubility and possibly prevents αS proteasomal degradation.
USP8	Counteracts NEDD4- dependent K63 polyUb. Inhibits αS endosomal degradation and increases endogenous αS level particular in the soma region of iPSC neurons.
USP13	Stabilizes αS by possibly preventing its degradation. Degradation pathway remains unknown.
USP19	Promotes αS secretion through misfolding associated protein secretion (MAPS) and reduces αS ubiquitination to facilitate aggregate formation.

## 2 Ubiquitination

### 2.1 Overall structure and functions

Ubiquitin is a highly conserved 76 amino acid peptide found in all eukaryotes. In humans, it is encoded by four genes: UBB, UBC, UBA52, and RPS27A (Lenkinski et al., 1977; Vijay-Kumar et al., 1987), each of which encodes either polyubiquitin or ubiquitin-fused to a ribosomal subunit (Finley et al., 1989; Redman and Rechsteiner, 1989). The polyprotein products of these genes are co-translationally cleaved by deubiquitinases (DUBs) to generate free ubiquitin.

Ubiquitin exerts almost all its functions by being conjugated to other proteins. Ubiquitin has a *β*-grasp globular structure and a flexible C-terminal tail of 6 residues (Vijay-Kumar et al., 1987). The globular surface includes recognition patches such as the hydrophobic patch formed by Leu8, Val70, and Ile44, which is recognized by most ubiquitin-binding domains (UBDs) and the ubiquitin receptors on the 26S proteasome (Dikic et al., 2009). Ubiquitin covalently attaches to substrates via its C-terminal Gly76, most commonly to the *ε*-amino group of the side chain of lysine residues in the target protein to form an isopeptide bond. Occasionally, it can also be conjugated to the side chain thiol or hydroxyl group of Cys, Ser, and Thr residues or to the N-terminal amino group of the protein (Cadwell and Coscoy, 2005; Ishikura et al., 2010; Shimizu et al., 2010; Trausch-Azar et al., 2010; Wang et al., 2007; Williams et al., 2007). Additionally, ubiquitin can also be conjugated onto one of the seven lysine residues (Lys6, Lys11, Lys27, Lys29, Lys33, Lys48, and Lys63) or N-terminal Met1 of the ubiquitin already attached to the target protein and the repetition of this reaction results in the formation of a polyubiquitin chain (Komander and Rape, 2012).

**Figure 1:**
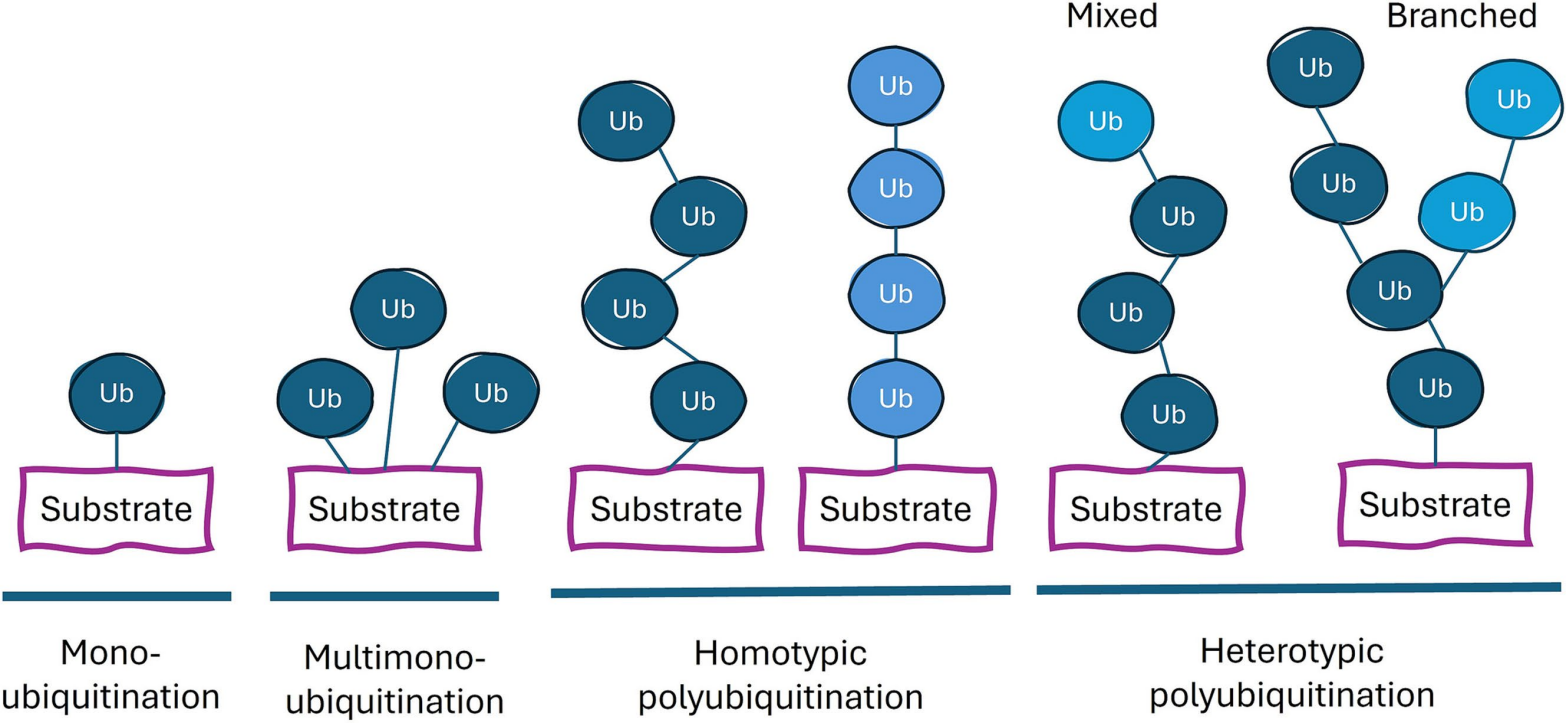
Types of ubiquitin linkages. Proteins can be conjugated with a single ubiquitin (monoubiquitination), or a ubiquitin on multiple lysine residues (multi-monoubiquitination) or with a chain of ubiquitin moieties (polyubiquitination). The color of ubiquitin (dark blue or light blue) indicates different types of linkage. Each ubiquitin in the chain can be conjugated to the more proximal ubiquitin at the latter’s M1, K6, K11, K27, K29, K33, K48, or K63 residue. Homotypic chains employ only one type of linkage. Heterotypic polyubiquitination contains at least two different types of ubiquitin linkages and potentially combine multiple chain types within a single polyubiquitin structure.

The many different possible products of the above ubiquitination reaction and their molecular and cell biological consequences has been referred to as the ubiquitin code. These different types of ubiquitination can be categorized based on the number and arrangement of the ubiquitins attached to the substrate: monoubiquitination (a single ubiquitin on one residue of the substrate protein), multi-monoubiquitination (single ubiquitins on multiple residues), and polyubiquitination (a chain of ubiquitins on one or more residues; Figure 1). Polyubiquitination can be further divided into homogeneous chains (single linkage type) and heterogeneous chains (multiple linkage types), leading to mixed or branched chains (Dongdem et al., 2024). Different linkages result in distinct chain conformations, allowing this code to be deciphered through the binding of different proteins to these distinct structures of ubiquitin on the target protein. This complexity is further enhanced by post-translational modifications of ubiquitin such as phosphorylation, acetylation and ADP-ribosylation (Song and Luo, 2019). In addition to recruiting other proteins, the ubiquitination of a protein can alter the latter’s structure and/or its binding partners, thereby influencing the latter’s biological functions.

The earliest and best described function of ubiquitination is its ability to target its conjugated protein for degradation by the proteasome. Classically, Lys48 polyubiquitin chains target proteins substrates to the proteasome, but it is now apparent that other chain types can also do so. Subsequently, ubiquitination of proteins – in particular with Lys63 chains - was shown to target to lysosomal proteolysis via autophagy (Shaid et al., 2013) or endosomes (Urbé, 2005). Other processes regulated by ubiquitination include immune activation, DNA damage response, and vesicular trafficking and some of these employ linear polyubiquitination or monoubiquitination as the signal (Chen and Sun, 2009; Clague et al., 2019; Komander and Rape, 2012; Schnell and Hicke, 2003). In this review, we will focus on functions of ubiquitin that are most relevant to αS homeostasis in PD.

### 2.2 The ubiquitination process

The ubiquitin code is generated through the coordinated action of three enzymes: E1 (ubiquitin-activating enzyme), E2 (ubiquitin-conjugating enzyme), and E3 (ubiquitin ligase). E1 activates ubiquitin in an ATP-dependent process and transfers the activated ubiquitin to E2. E2, in conjunction with E3, facilitates ubiquitin conjugation to the substrate, with E3 providing substrate specificity. Both E2 and E3 enzymes influence the type of ubiquitin linkage formed.

Humans have two E1 genes, approximately 40 E2s and about 600 E3s (Li et al., 2008). E3s can be categorized into three families: RING (Really Interesting New Gene), HECT (Homologous to E6AP Carboxyl Terminus), and RBR (RING-between-RING).

RING E3s comprise approximately 95% of ligases, can be single or multi-subunit complexes. They act as scaffolds, bringing together the substrate and ubiquitin-charged E2 enzyme which binds to the Zn containing RING finger domain on the E3. Within RING E3 mediated polyubiquitination, linkage specificity is determined by the particular E2 enzyme recruited (Ye and Rape, 2009). U-box type RING E3s have a similar RING finger structure but without coordinated Zn atoms. Cullin-RING and APC/C type ligases are examples of multi-subunit E3 complexes in which one of the subunits contains the RING finger domain and another the substrate recognition domain. HECT E3 ligases have a catalytic cysteine residue at their C-terminal lobe, which directly receives ubiquitin from E2 enzymes, thus determining linkage specificity independently of the E2 partner. Among HECT E3 ligases are the Nedd4 and HERC subclasses, members of which share conserved domains N-terminal to the catalytic HECT domain (Rotin and Kumar, 2009). RBR E3s use a hybrid RING/HECT mechanism, featuring three Zn-binding domains: RING1, in-between RING (IBR), and RING2. RING1 binds the E2-Ub complex like RING E3s, while RING2 contains a catalytic cysteine that transfers ubiquitin from E2 to the E3, similar to HECT E3s.

### 2.3 Deubiquitination

Ubiquitination, like all post-translational modifications, is reversible. In addition to processing newly synthesized ubiquitin, deubiquitinases (DUBs) counteract ubiquitination by cleaving ubiquitin from substrates. In mammalian cells, more than half of the ubiquitins found are monoubiquitinated, often on histones, with the remainder forming chains (~15%) or existing freely (~20%) (Kaiser et al., 2011). There are ~100 identified DUBs, divided into seven families: Ubiquitin-Specific Proteases (USPs), Ubiquitin C-terminal Hydrolases (UCHs), Ovarian Tumor Proteases (OTUs), Josephin Domain DUBs (MJDs), Motif Interacting with Ubiquitin-N-terminal Y domain (MINDY), Zinc finger with UFM1-specific peptidase domain protein (ZUFSP), and JAMM/MPN. All DUB families are cysteine proteases, except JAMM/MPN, which are Zn-dependent metalloproteinases (Clague et al., 2019). Although some DUBs have broad specificity, many are selective and achieve this selectivity by targeting specific substrates through protein interaction domains or recognizing specific ubiquitin chain architectures. In the latter case, the end product is usually a monoubiquitinated substrate. Linkage selectivity is determined either by the catalytic domain of the DUBs or their ubiquitin-binding domains (UBDs) or associated proteins (Mevissen and Komander, 2017).

## 3 A-synuclein

### 3.1 Biochemical features

**Figure 2:**
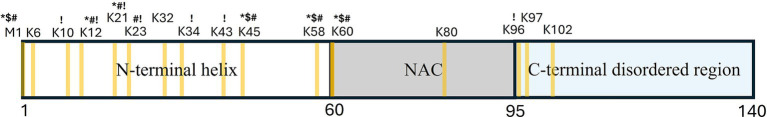
Schematic diagram of α-synuclein structure, illustrating its division into the N-terminal helix, non-amyloid-β component (NAC) domain, and C-terminal disordered region. Lysine and M1 residues potentially involved in ubiquitination are highlighted in yellow. Ubiquitination sites confirmed in different models are labeled as follows: *-cell-lines; $-primary mice neurons; #-human samples; !-cell free assay.

αS is a 14 kDa protein, comprising three regions: (1) an N-terminal helix (residues 1–60) required for lipid-binding (Davidson et al., 1998; Eliezer et al., 2001), (2) a central non-amyloid-*β* component (NAC; residues 61–95) essential for aggregation (Giasson et al., 2001; Uéda et al., 1993), and (3) a C-terminal disordered region (residues 96–140) that interacts with ions and promotes SNARE complex assembly (Burré et al., 2010; Figure 2). Recent studies have revealed overlapping functions of these regions, suggesting the importance of intra- and inter-molecular interactions between them (Doherty et al., 2020; Fusco et al., 2014; Fusco et al., 2016; Ulamec et al., 2022).

ɑS is part of a protein family that includes beta-synuclein (βS) and gamma-synuclein (γS), each encoded by distinct genes (George, 2002) Phylogenetic studies show that ɑS and βS are more closely related to each other than to γS (Siddiqui et al., 2016). All synucleins share a highly conserved N-terminal sequence and are mainly expressed in the brain according to the Human Protein Atlas. Synuclein orthologues are absent in invertebrates, consistent with their crucial role in the central nervous system of higher organisms. The ɑS protein sequence is highly conserved between humans and rodents, differing by only six amino acids.

Initially considered a cytosolic protein with an intrinsically disordered monomeric structure, subsequent cross-linking studies indicate that αS exists predominantly as a tetramer. This tetramer has a compact structure shielding the NAC region, preventing spontaneous aggregation (Bertoncini et al., 2005; Kang et al., 2012; Theillet et al., 2016). The tetrameric structure is in equilibrium with the disordered monomer and dependent on αS concentration (Bartels et al., 2011; Wang et al., 2011). Furthermore, the tetramer can be formed upon membrane binding where it becomes functional for SNARE complex assembly (Burré et al., 2014). αS disease causing mutations such as A53T, E64K and G51D, alter the tetrameric/monomeric ratio and are associated with neuronal toxicity (Dettmer et al., 2015a; Dettmer et al., 2015b; Nuber et al., 2018; Nuber et al., 2024).

However, the existence of a stable tetrameric form has been challenged, as the tetrameric species have failed to be detected by other groups (Burré et al., 2013; Corbillé et al., 2016; Fauvet et al., 2012). These opposing views highlight the ongoing debate in the field, with some arguing that αS exists in multiple equilibrium states, varying between monomers, tetramers, and other higher-order oligomers depending on cellular and environmental contexts (Burré et al., 2014; Fauvet et al., 2012).

### 3.2 Physiological functions

The most prominent physiological function for αS is its role in synaptic vesicle (SV) regulation due to its localization at the presynaptic terminal (Maroteaux et al., 1988) and its affinity for high-curvature membranes (Davidson et al., 1998). During synaptic transmission, calcium influx prompts the recruitment of SVs to the presynaptic active zone, where they fuse with the presynaptic membrane to release neurotransmitters. Fluorescence resonance energy transfer assays suggest that ɑS acts as a SNARE complex chaperone by binding to phospholipid membranes and multimerizing at the presynaptic membrane (Burré et al., 2014; Burré et al., 2010; Lou et al., 2017). An interaction between VAMP2 (synaptobrevin-2) and the ɑS C-terminus at the docking site allows ɑS to promote SV exocytosis by preventing SV fusion pore closure (Logan et al., 2017). Overexpression of ɑS restricts neurotransmitter release by clustering SVs, a process requiring ɑS multimerization and association with VAMP2 and synapsin (Larsen et al., 2006; Nemani et al., 2010). Phosphorylation of ɑS at S129, a marker of pathological ɑS aggregates, enhances this clustering (Parra-Rivas et al., 2023), inhibiting SV movement to the presynaptic active zone and reducing synaptic transmission (Atias et al., 2019; Diao et al., 2013; Sun et al., 2019). Besides these critical functions at the SV, recent research has also suggested roles for αS in regulating mRNA stability (Hallacli et al., 2022), gene transcription (Davidi et al., 2020), and DNA stability (Vasquez et al., 2020).

### 3.3 Pathophysiology of αS: from monomer to aggregates

The mechanisms by which soluble monomers or tetramers of αS become converted into insoluble fibrils mostly localized at the neuronal soma remain unclear. This is partly due to the long time required for fibril development *in vivo* and the underexplored protein–protein interactions of native ɑS. Recent research highlights the dynamic interaction of ɑS with its paralogues, *β*S and γS, at SVs (Carnazza et al., 2022). These studies show that the equilibrium between cytosolic ɑS monomers and SV-binding multimers is regulated by ɑS/βS and ɑS/γS heteromers. Specifically, heteromer formation inhibits ɑS from binding to SVs, and decreases the cytosolic concentration of unfolded monomeric ɑS, which might be aggregation-prone (Bartels et al., 2011; Wang et al., 2011).

Maintaining normal levels of ɑS is crucial, as multiplication of the SNCA gene encoding ɑS can induce PD pathology (Ibáñez et al., 2004; Polymeropoulos et al., 1997; Singleton et al., 2003). Although ɑS knockout has little effect on synaptic function and neuronal survival (Abeliovich et al., 2000; Chandra et al., 2005), ɑS is essential under stressful conditions, such as the loss of the synaptic protein CSPɑ/DNAJC5 (Chandra et al., 2005; Greten-Harrison et al., 2010).

Cell free biophysical studies suggest that the formation of ɑS amyloid fibril containing stable parallel β-sheet structures arises from a nucleation-polymerization process (Cremades et al., 2012; Iljina et al., 2016). During primary nucleation, soluble monomers nucleate to generate oligomers. Initially formed oligomers are transient and can rapidly undergo secondary polymerization to form fibrils or convert into more kinetically stable and toxic oligomers, potentially causing cell membrane damage (Fusco et al., 2017; Kayed et al., 2003). Fragmented fibrils can also act as seeds to promote secondary nucleation (Cohen et al., 2011). Oligomers, with a greater surface-to-volume ratio, show higher toxicity in cells compared to fibrils (Chen et al., 2015; Cremades et al., 2012). However, in contrast to consistent fibrillar structures, oligomers are a mixture of heterogeneous structures with variabilities in size, β-sheet content, and toxicity (Chen et al., 2015; Cremades et al., 2012; Gallea and Celej, 2014). Increased membrane damage by oligomers could be due to antiparallel β-sheet content which exposes hydrophobic side chains that interact with cellular lipids (Celej et al., 2012; Chen et al., 2015).

Interestingly, *in vivo* studies show that injection of fragmented fibrils are more toxic than non-fragmented ones or oligomers in mice brains (Froula et al., 2019). The increased seeding capacity of these fragmented fibrils suggests that aggregate propagation and formation —whether through oligomer polymerization or fibril fragmentation—play a critical role in the pathogenesis of PD. Research in cellular systems demonstrate that oligomers released from fibrils cause neuronal dysfunction, while stable fibrils can prevent ɑS toxicity (Cascella et al., 2021; Lam et al., 2016). These findings highlight the importance of oligomer dynamics during aggregate formation in PD (Conway et al., 2000; Mahul-Mellier et al., 2020; Mahul-Mellier et al., 2015).

Other factors, such as lipid vesicles and pH, also strongly affect aggregate formation (Buell et al., 2014; Flagmeier et al., 2016; Galvagnion et al., 2015; Grey et al., 2011; Kumari et al., 2021; Lee et al., 2005). *In vitro*, small unilamellar vesicles (SUVs) promote ɑS primary nucleation, contributing to amyloid fibril formation (Galvagnion et al., 2015). The secondary nucleation process is promoted by a low pH environment, suggesting that endo-lysosomal vesicles with acidic microenvironments in cells may play a key role in aggregate formation (Buell et al., 2014; Kumari et al., 2021).

The discovery of ɑS in cerebrospinal fluid (El-Agnaf et al., 2003) and LBs in fetal grafted neurons of Parkinson’s patients (Kordower et al., 2008; Li et al., 2008) support a prion-like behavior. Studies show that exogenous ɑS preformed fibrils (PFFs) can induce LB-like structures in cultured cells and *in vivo*, promoting ɑS fibril elongation (Luk et al., 2012; Luk et al., 2009; Volpicelli-Daley et al., 2011). Endogenous ɑS is required for fibril formation, and these fibrils can spread from injection sites, even outside the CNS such as the gut, indicating inter-neuronal propagation (Ayers et al., 2017; Macdonald et al., 2021; Osterberg et al., 2015). However, the exact mechanisms and the nature of ɑS species responsible for this spreading remain largely unknown, raising questions about how ɑS is released and taken up.

## 4 Characteristics of ubiquitinated *α*-synuclein in Lewy bodies

αS is the main protein component of LBs, the pathological hallmark of PD (Spillantini et al., 1997), LBs have been known to stain with anti-ubiquitin antibodies since the late 1980s, well before αS was identified as the major protein component (Baba et al., 1998; Kuzuhara et al., 1988; Lennox et al., 1989; Spillantini et al., 1997). Initially, ubiquitin staining was observed at the peripheral rim of LBs (Kuzuhara et al., 1988). However, multiplex confocal imaging revealed that ubiquitin is either colocalized with *α*S in less compact LBs or present in the center of more compact LBs and partially colocalized with αS, which is stained mainly at the peripheral shell (Gai et al., 2000). The discovery of C-terminal truncated αS at Asp119/Asn122 (Anderson et al., 2006; Baba et al., 1998; Dufty et al., 2007; Li et al., 2005) and phosphorylated α-S (p129-αS) (Fujiwara et al., 2002) as the predominant αS species in LBs, coupled with techniques such as three-dimensional reconstruction, multicolor antibody labeling, and high-resolution stimulated emission depletion (STED) imaging have led to further elucidation of the intricate structure of classical concentric LBs. Altogether, these data suggest a model where αS conjugates with ubiquitin and recruits non-ubiquitinated αS, maturing into a compact LB structure. Ubiquitin and C-terminal truncated αS are found in the core, p129 or full-length αS in the middle layer, and neurofilaments and cytoskeletal proteins in the outer layer of LBs (Kanazawa et al., 2008; Moors et al., 2021; Prasad et al., 2012).

Although imaging studies revealed co-localization of ubiquitin and αS, it was studies using two-dimensional gel electrophoresis that identified αS as the primary ubiquitinated protein in LBs. In these studies, the shifted αS (Syn-1 antibody-positive bands) corresponded with anti-ubiquitin antibody staining (Anderson et al., 2006; Tofaris et al., 2003). Interestingly, ubiquitinated αS was primarily associated with p129-αS species, which constitute more than 90% of the insoluble αS species in dementia with Lewy bodies (DLB) brains, and are modified by mono-, di-, or, to a lesser extent, poly-ubiquitin (Anderson et al., 2006; Hasegawa et al., 2002; Tofaris et al., 2003). Mass spectrometry analysis identified three ubiquitination sites on αS: Lys12, Lys21, and Lys23 (Anderson et al., 2006). However, the biological consequences and regulatory mechanisms of these modifications remain largely unexplored.

## 5 Functions of ubiquitination of α-synuclein

### 5.1 Ubiquitination and intracellular proteolytic systems

**Figure 3:**
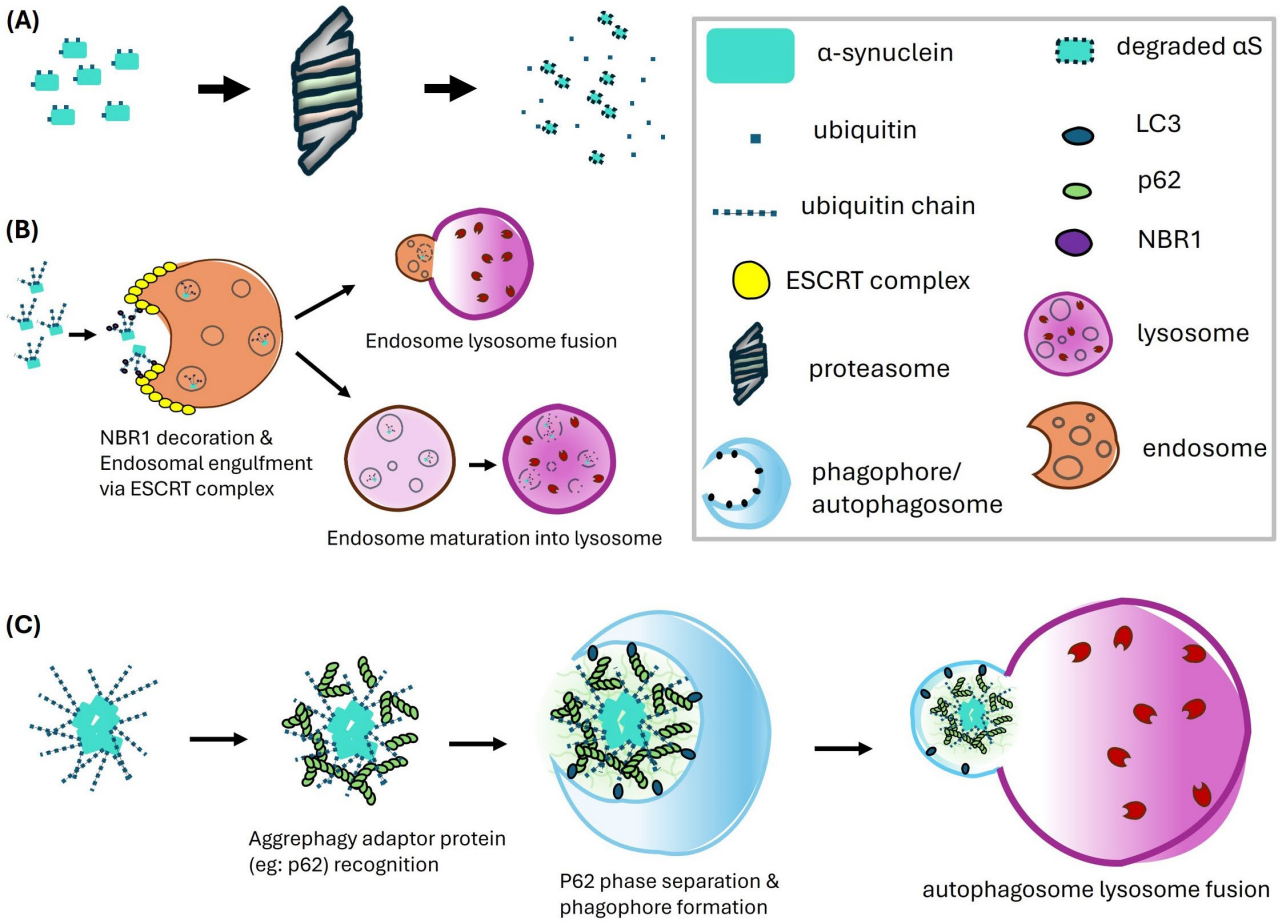
Proteolytic pathways of α-synuclein mediated by ubiquitination, including the proteasome pathway **(A)**, the endosome-lysosome pathway **(B)**, and the macroautophagy-lysosome pathway **(C)**. **(A)** In the proteasome pathway, α-synuclein undergoes ubiquitination which targets the protein for recognition and degradation by the proteasome. **(B)** The endosome-lysosome pathway has emerged as a major route for α-synuclein degradation, predominantly involving K63-linked polyubiquitination. The ubiquitinated α-synuclein is recognized by NBR1, which may interact with the ESCRT complex to facilitate endosomal engulfment and the formation of multivesicular bodies (MVBs). The endosomes carrying α-synuclein either fuse with lysosomes or follow a maturation pathway, where a gradual decrease in pH activates proteolytic enzymes, ultimately leading to the degradation of α-synuclein. **(C)** The macroautophagy-lysosome pathway primarily targets polyubiquitinated α-synuclein aggregates. Ubiquitin chains on these aggregates are recognized by aggrephagy adaptor proteins, such as p62. The polymerization of p62 facilitates phase separation of the sequestered aggregates and serves as a platform for interaction with LC3, aiding in phagophore formation. The autophagosome containing the aggregates then fuses with the lysosome, where degradation occurs.

Ubiquitination is involved in both major intracellular proteolytic systems - the proteasome and lysosome (Figure 3). The latter receives cargo through several pathways, in particular through autophagy and endosomes. Autophagy can be further divided into macroautophagy (MA), chaperone-mediated autophagy (CMA), and microautophagy. Below, we briefly introduce the mechanisms of these protein degradation pathways, which have been reviewed in detail elsewhere (Clague and Urbé, 2010; Collins and Goldberg, 2017; Finley, 2009).

#### 5.1.1 Proteasome

The proteasome was the first proteolytic machinery linked to the ubiquitination system, establishing the connection between ubiquitination and protein homeostasis (Waxman et al., 1987). The proteasome consists of two main components: the 20S core particle (CP) and the 19S regulatory particle (RP). The CP contains the proteolytic site, with a barrel-shaped structure composed of α7β7β7α7 heteroheptameric rings of subunits. Among these, the β1, β2, and β5 subunits are responsible for proteolytic cleavage with each subunit having different amino acid specificities for cleavage. The entrance of the lumen of the free CP is closed by the N-terminal tails of the α subunits but opens when the RP caps the CP to form the holoenzyme.

The RP recognizes ubiquitinated proteins, removes the ubiquitin chains, and unfolds the proteins for entry into the narrow CP channel for degradation. This recognition requires either direct binding of the ubiquitin chain on the substrate to ubiquitin receptor subunits of the RP or indirect binding of the ubiquitin to intermediary shuttling factors such as Ddi1, Dsk2, and Rad23. These factors contain both ubiquitin-associated (UBA) domains which associate with ubiquitinated substrates and ubiquitin-like (UBL) domains which bind the RP. Both direct and indirect bindings involve RP ubiquitin receptors such as PSMD4/Rpn10, ADRM1/Rpn13, and PSMD2/Rpn1, which use ubiquitin-interacting motif (UIM), Pleckstrin-like receptor for ubiquitin (Pru) domain, and T1 site, respectively to bind ubiquitinated substrates (Shi et al., 2016).

After the initial binding of ubiquitinated substrates, a tight-binding step occurs in an ATP-dependent manner (Peth et al., 2010). This transition step allows a loosely folded region of the substrate to be tightly bound to the ATPase ring of the RP for subsequent unfolding and translocation into the CP for degradation (Prakash et al., 2004). Before degradation, ubiquitin chains are removed and released, enabling substrate passage through the CP channel and generating free ubiquitin for reuse. This process is regulated by proteasome-associated DUBs, including PSMD14/Rpn11, USP14/Ubp6, and UCH37/UCHL5. These DUBs may also act as proofreading mechanisms to release substrates not committed to proteasome degradation (Crosas et al., 2006; Jacobson et al., 2009; Lu et al., 2015).

PD has been linked to proteasome machinery since proteasomes were identified in LBs (Ii et al., 1997). αS has been confirmed to be degradable by the proteasome, with the familial A53T mutant being degraded less efficiently (Bennett et al., 1999). *In-vitro* studies have shown that monoubiquitinated αS has a higher degradation rate by the proteasome compared to free αS, with ubiquitination at Lys6, Lys12, Lys21, and Lys32 having the strongest effect (Abeywardana et al., 2013; Shabek et al., 2012). However, whether the proteasome is impaired in PD remains controversial, as functionally impaired forms have been reported (McNaught et al., 2002; McNaught and Jenner, 2001). Tofaris et al. demonstrated that proteasome function is impaired specifically in the substantia nigra in PD brains, while proteasomal function remains intact in other brain regions. Importantly, this regional deficit in proteasomal activity was not associated with a corresponding regional accumulation of ubiquitinated αS, suggesting that proteasome impairment is not associated with ubiquitinated αS accumulation (Tofaris et al., 2003).

#### 5.1.2 Macroautophagy

Like the ubiquitin-proteasome system (UPS), components of the autophagy machinery have also been discovered in LBs of PD patients by immunohistochemistry (Alvarez-Erviti et al., 2010; Dehay et al., 2010). Several PD risk genes associated with autophagy-lysosome pathways (ALP) such as LRRK2, ATP13A2 and VPS35 have been identified in genome-wide association studies (GWAS) (Chang et al., 2017; Nalls et al., 2014). While these genes are associated with ALP, they also have broader effects on endolysosomal function, such as regulating endosomal trafficking and membrane dynamics, which may indirectly influence autophagy. Additionally, enzymatic activities of lysosomal proteins are decreased in PD patients (Moors et al., 2019). These findings suggest that ALP plays an important role in αS homeostasis in PD.

In MA, the autophagosome, a double-membrane structure decorated with LC3/ATG8, engulfs targeted cargoes before fusing with lysosomes for degradation. The selectivity of ubiquitinated cargoes is achieved through intermediary autophagy receptors. These receptors contain both ubiquitin-binding domains (UBDs) and LC3-interacting region (LIR) motifs, allowing them to bridge ubiquitinated proteins and autophagosomes (Lamark and Johansen, 2012).

MA plays a significant role in the clearance of protein aggregates, unlike proteasomes, which target single protein molecules for unfolding (Pohl and Dikic, 2019). The selective removal of protein aggregates by autophagy is termed aggrephagy (Øverbye et al., 2007). Numerous aggrephagy receptors have been identified, including p62/SQSTM1 (Janssen et al., 2018; Komatsu et al., 2007; Lim et al., 2015; Matsumoto et al., 2011; Pankiv et al., 2007; Tanji et al., 2015; Zellner et al., 2021), NBR1 (Nicot et al., 2014; Odagiri et al., 2012; Rué et al., 2013; Zellner et al., 2021), OPTN/optineurin (Korac et al., 2013; Osawa et al., 2011; Shen et al., 2015; Zellner et al., 2021), ALFY (Clausen et al., 2010; Filimonenko et al., 2010), Tollip (Lu et al., 2014; Zellner et al., 2021), TAX1BP1 (Sarraf et al., 2020; Turco et al., 2021), and CCT2 (Ma et al., 2022; Pavel et al., 2016). Among these, p62 (Mahul-Mellier et al., 2020; Tanji et al., 2015; Watanabe et al., 2012), NBR1 (Odagiri et al., 2012), TAXBP1 (Lannielli et al., 2022), and Tollip (Chen et al., 2017) have been shown to directly associate with *α*S for MA turnover.

p62 has been characterized since its early identification in LBs (Kuusisto et al., 2003) and is well-studied for its role in ubiquitination and aggrephagy. Aggrephagy receptors typically prefer K63-linked over K48-linked polyubiquitin chains on substrates (Kirkin et al., 2009; Long et al., 2008; Matsumoto et al., 2011; Seibenhener et al., 2004; Sun et al., 2018; Wurzer et al., 2015; Zaffagnini et al., 2018), though some show better affinity for Met1-linked linear ubiquitin chains (Wurzer et al., 2015). The molecular mechanism of aggrephagy involves the binding of ubiquitinated aggregates, requiring p62 clustering, polymerization, and phase separation (Sun et al., 2020). K48 chains can disrupt this process (Ciuffa et al., 2015; Sun et al., 2018; Wurzer et al., 2015; Zaffagnini et al., 2018). These protein clusters act as platforms for autophagosome formation, which eliminates ubiquitinated aggregates by fusing with lysosomes (Agudo-Canalejo et al., 2021; Kageyama et al., 2021). Interestingly, this clustering process may also facilitate the formation of protein inclusions. Studies on K63 polyubiquitination have shown that overexpression of ubiquitin specific for K63 linkage accelerates the formation of protein inclusions, while a K63R mutant construct inhibits this process (Lim et al., 2005; Olzmann et al., 2007; Tan et al., 2008; Wang et al., 2012). These findings highlight an important role for ubiquitination and MA in the formation of LBs.

#### 5.1.3 Chaperone-mediated autophagy

CMA is discovered as a lysosomal degradation pathway for αS in rat ventral midbrain cultures, as suggested by significant inhibition of degradation by lysosomal inhibition (ammonium chloride) and minimal impact from macroautophagy (3-methyladenine) and proteasome (epoxomicin) inhibition (Cuervo et al., 2004). In CMA, the Hsc70 chaperone recognizes the KFERQ-like motif on αS (95VKKDQ99) independently of ubiquitination and forms a complex with it. This complex associates with the lysosomal membrane receptor LAMP2A, the rate-limiting component of CMA, which forms a multimeric protein complex to facilitate αS translocation into the lysosomal lumen. Interestingly, αS A53T and A30P mutants block this translocation by tightly binding to LAMP2A receptors (Cuervo et al., 2004; Xilouri et al., 2009), suggesting the importance of CMA in αS homeostatic clearance. CMA efficiency decreases with aging, and mutations in PD risk genes such as LRRK2 can interfere with CMA by blocking the LAMP2A multimerization required for translocation (Ho et al., 2020; Kabuta et al., 2008; Orenstein et al., 2013; Xilouri et al., 2009), resulting in αS accumulation on the lysosomal membrane and accelerating αS oligomerization (Ho et al., 2020; Orenstein et al., 2013). However, since CMA substrate translocation depends on their unfolding, similar to the proteasome pathway, it is unlikely that CMA is directly involved in clearing αS aggregates.

#### 5.1.4 Microautophagy and endosomal pathways

Microautophagy, initially studied in yeast, involves lysosomes directly engulfing targeted substrates (Wang et al., 2023). Recently, endosomal microautophagy (eMI) has been discovered in mammalian cells, where targeted substrates are delivered to late endosomes before fusing with lysosomes (Sahu et al., 2011). Unlike CMA, eMI can remove substrates without requiring their unfolding; therefore, eMI can target protein clusters (Sahu et al., 2011). eMI selectively uses Hsc70 for substrate recognition (Morozova et al., 2016; Sahu et al., 2011), but does not depend on LAMP2A receptors for translocation. Instead, it relies on the ESCRT systems and Hsc70 interactions with endosomal membranes to internalize cargo into late endosomes, forming multivesicular bodies (MVBs) for lysosomal degradation (Sahu et al., 2011). The degradation of αS via endosomal pathways, where αS is trafficked to endosomes before lysosomal degradation (Boassa et al., 2013; Gerez et al., 2019; Sugeno et al., 2014; Tofaris et al., 2011; Zenko et al., 2023) has also been reported. However, the uptake of αS degradation in this endosomal pathway does not require Hsc70 binding. Instead, NEDD4 catalyzes K63-polyubiquitination of αS which allows it to associate with the ESCRT complex for endosomal uptake (Tofaris et al., 2011, Zenko et al., 2023).

## 6 Enzymes regulating ubiquitination of α-synuclein

### 6.1 Neural precursor cell expressed developmentally down-regulated protein 4

NEDD4-1 is a member of the NEDD4 subset of HECT E3s (Rotin and Kumar, 2009). These E3s contain WW domains that recognize PY motifs (PPxY or LPxY amino acid sequences) or phosphorylated serine/threonine residues in target proteins. NEDD4 is the most studied E3 in αS ubiquitination, recognizing αS through the interaction of its WW domain with the C-terminal proline-rich region 120-(PDNEAYEMPSEEGY)-133 (Tofaris et al., 2011). αS is co-immunoprecipitated with NEDD4 from both mouse and human brain lysates and colocalized with LBs in pigmented neurons (Tofaris et al., 2011).

NEDD4 polyubiquitinates αS with mainly K63 chain linkages. This leads to αS degradation via endosomal pathways as knocking down the ESCRT III core component VPS24 or the ESCRT I core component TSG101 blocked NEDD4-dependent αS degradation (Tofaris et al., 2011). This conclusion is also supported by studies in which SH-SY5Y cells were exposed to recombinant αS oligomers (~40 kDa) in the medium (Sugeno et al., 2014). The oligomers accumulated within endolysosome vesicles, including Rab7 (late endosome) and lysotracker (lysosome) positive vesicles. This internalization into endosomes was promoted by NEDD4 ubiquitination of membrane associated αS. Overexpression of a catalytic mutant (C867A) and ΔWW form of NEDD4 decreased its ability to sort αS to endosomes compared to wild-type NEDD4. Knocking down the ESCRT III component CHMP2B prevented the NEDD4-dependent αS degradation confirming the trafficking through endosomes.

Besides these effects on αS monomers and oligomers, Mund et al. demonstrated using cell free assays that most NEDD4 family ligases prefer to polyubiquitinate αS fibrils over monomers, with NEDD4-1 showing the strongest ubiquitination capacity. This linkage type appeared to be K63, based on cleavage susceptibility by deubiquitinases with varying specificities (Mund and Pelham, 2018). Deletion analysis of the C2 domain of NEDD4 decreased its ability to polyubiquitinate αS fibrils, while further deletion of the WW domain had a minor effect, suggesting NEDD4 binds αS fibrils via the C2 domain rather than WW-PY interaction (Mund and Pelham, 2018).

The role of NEDD4 in handling aggregates in cells was tested in HEK293 cells expressing αS fused to split halves of Venus fluorescent protein and aggregates detected as punctate bimolecular fluorescence complementation (BiFC) signals. Knocking down NEDD4 in these cells showed that αS ubiquitination occurs mainly at αS K45/58/60 residues via K63 poly-ubiquitin chains (Zenko et al., 2023). This ubiquitination is essential for αS turnover, mediated by the aggrephagy receptor NBR1, but not by p62, OPTN, or TAX1BP1. Knocking down autophagy core protein ATG7 or CMA/eMI core protein HSC70 did not affect αS turnover, but knocking down ESCRT components (TSG101, CHMP3) did, highlighting the role of an endosomal mediated pathway for degradation.

An antibody raised against αS conjugated with di-Gly peptides at the K45/58/60 ubiquitination sites was confirmed to predominantly recognize αS ubiquitinated by NEDD4 and BiFC puncta in the cells. This antibody colocalized with ubiquitin in LBs from PD brains and the autophagy adaptor protein NBR1, as well as with lysosomal marker LAMP1+ vesicles, but not with autophagosome marker LC3II in primary neurons. This supports the role of NBR1 in the endosomal degradation pathway of K45/58/60-ubiquitinated αS in the neurons (Zenko et al., 2023). NEDD4’s effects on αS ubiquitination are also indirectly regulated by the DUB YOD1, which stabilizes NEDD4 in a dose-dependent manner (Park et al., 2023), and by the SUMO enzyme PIAS2 which by SUMOylating αS inhibits NEDD4-mediated αS ubiquitination (Rott et al., 2017). *In vivo*, NEDD4 can rescue PD-associated pathology. AAV expression of A53T αS in rat substantia nigra causes dopaminergic neuron loss and increases αS aggregates. These pathologies can be reduced by co-injection of AAV expressing wild-type NEDD4, but not a catalytic mutant (Davies et al., 2014). Taken together, these observations underscore NEDD4’s role as a prominent E3 ligase that can target αS for lysosomal degradation via endosomes.

### 6.2 Seven *in absentia* homolog 1 and 2 and USP9X

SIAH-1 and SIAH-2 are RING-type E3s initially identified for their interaction with synphilin-1, a protein associated with αS in LBs (Engelender et al., 1999; Liani et al., 2004; Wakabayashi et al., 2000). Later immunohistochemical staining also identified SIAH proteins in LBs (Liani et al., 2004). Both SIAH-1 and SIAH-2 can promote ubiquitination of αS in cells, but SIAH-2 appears to have stronger affinity for αS and ubiquitinates it more effectively in a cell free system (Liani et al., 2004; Rott et al., 2008). Mass spectrometry analysis identified several ubiquitination sites on αS mediated by SIAH-2 in a cell free assay, including K10, K12, K21, K23, K34, K43, and K96 (Rott et al., 2008). These findings align with previous reports of αS ubiquitination sites at K12, K21, and K23 observed in LBs (Anderson et al., 2006). Notably, even with two-thirds of these ubiquitination sites abolished, αS ubiquitination by SIAH-2 persisted, demonstrating the flexibility of the ubiquitination process (Hanna et al., 2007; Kim et al., 2011). Neither enzyme promotes polyubiquitination, but inhibitors of autophagy-lysosome system (ALS) or the proteasome have been observed to stabilize αS following overexpression of SIAH2 (Rott et al., 2008), suggesting that these ubiquitinated forms of αS do target for degradation.

Other studies have indicated that SIAH isoforms mediated monoubiquitination promotes formation of insoluble aggregates both in cells and cell free systems (Lee et al., 2008; Liani et al., 2004). In addition, silencing of the deubiquitinase USP9X which counteracts SIAH-2-dependent αS monoubiquitination in SH-SY5Y cells increases αS aggregation and cytotoxicity under proteolytic (lactacytstin, NH_4_Cl and 3-MA) inhibition. Under normal conditions without proteolytic inhibition, such silencing of USP9X facilitates αS degradation (Rott et al., 2011; Rott et al., 2017). In cell-free systems, αS monoubiquitinated by recombinant SIAH2 can be degraded by purified proteasome (Rott et al., 2011; Rott et al., 2017). USP9X expression is decreased in PD and DLB brains which would be consistent with the above proposed mechanisms. Overall, the consequences of SIAH-dependent αS monoubiquitination—whether leading to degradation or aggregation—and its regulation through deubiquitination require further exploration *in vivo* to verify their precise roles in PD.

### 6.3 The SKP1-CUL1-F box protein complex

The SCF complex E3s are a subset of the Cullin-RING ligase family and consist of the scaffold protein Cullin-1 (Cul1), adaptor protein S-phase Kinase-associated Protein 1 (SKP1), the ligase RING-box protein (RBX1), and various F-box proteins that bind substrates (Zheng et al., 2002). Immunohistochemistry staining confirmed that Cul1 and SKP1 are colocalized with αS in LBs from the brains of patients with PD and DLB. Proteome analysis of SH-SY5Y cells revealed that SCF components, particularly Cul1 and SKP1, are upregulated in response to exposure of the cells to detergent-resistant αS fibrils. The exogenous fibrils accumulated within endolysosomal structures marked by Rab5A and LAMP1, suggesting involvement of the endosomal degradation pathway. Prolonged exposure to the fibrils beyond 6 h caused vesicle rupture, leading to seeding of endogenous αS and formation of high molecular weight aggregates, confirmed by immunoblotting.

The release of the fibrils into the cytoplasm resulted in their ubiquitination. A siRNA screen targeting 31-box proteins in HeLa cells identified FBXL5 as the specific F-box protein responsible for the ubiquitination (Gerez et al., 2019). Knockdown of other components of the SCF complex also prevented the ubiquitination of the fibrils. Mass spectrometry analysis showed that SCF ubiquitinates the fibrils primarily at K45, 58, and 60, forming K48-K63 branched ubiquitin chains. Interestingly, these lysine residues are typically buried within the fibril core according to cryo-EM studies (Guerrero-Ferreira et al., 2019; Yang et al., 2022), raising questions about the accessibility of these residues for ubiquitination. Knockdown of Cul1 or SKP1 in SH-SY5Y, BV-2 (microglia-like cell line), and COS-7 cells inhibits the degradation of the fibrils, which involves both lysosomal and proteasomal pathways, as evidenced by effects of their respective inhibitors, bafilomycin A1 and MG132 (Gerez et al., 2019).

In transgenic mice expressing human αS, which spontaneously form αS aggregates, injection of the fibrils induces LB-like pathology. Injection of lentivirus silencing SKP1 increased αS inclusions in those brain regions compared to injection with control virus. In nontransgenic mice, silencing SKP1 or FBXL5 led to p129-αS aggregates spreading, indicating SCF-FBXL5 inhibits LB-like pathology initiation and propagation induced by extracellular αS seeds (Gerez et al., 2019). These results support an important role for the SCF complex in mediating the degradation of internalized αS fibrils and thereby inhibiting subsequent seeding and further propagation.

### 6.4 Linear ubiquitin chain assembly complex

LUBAC is an RBR E3 complex renown for regulating immune and inflammatory responses, particularly NF-κB signaling, through the formation of linear (Met1-linked) ubiquitin chains. LUBAC consists of three main components: Haem-oxidized IRP2 ubiquitin ligase-1 (HOIL-1 L), HOIL-1 Interacting Protein (HOIP), and SHANK-associated RH domain interacting protein (SHARPIN). HOIL-1 L contains a C-terminal RING domain functioning as an E3 ligase, while HOIP is an RBR E3 possessing a linear ubiquitin chain determining (LDD) domain crucial for synthesizing linear ubiquitin chains. SHARPIN stabilizes the LUBAC complex through its interaction with HOIL-1 L (Gao et al., 2023).

A key target of LUBAC is NEMO and the M1-linked ubiquitin (M1-Ub) chain on NEMO serves to recruit critical subunits of the IκB kinase complex to promote NFκB signaling. M1-Ub and NEMO colocalize with αS in LBs from PD patients, and M1-Ub colocalizes with p129-αS in primary neurons treated with A53T preformed fibrils (PFFs), suggesting LUBAC’s involvement in protein aggregation (Furthmann et al., 2023). In SH-SY5Y cells, exposure to αS PFFs results in αS aggregates that associate with LUBAC components (Furthmann et al., 2023). Overexpression of wild-type NEMO or HOIP decreased αS aggregates and this reduction in αS aggregates depends on the aggrephagy adaptor p62 binding to M1-ubiquitin chains and is inhibited by bafilomycin A1, indicating the involvement of autophagy. NEMO promoted p62 condensation at αS aggregates, facilitating aggrephagy. This important role for NEMO in clearance of aggregates is supported by observations in a patient with Incontinentia pigmenti due to expression of a C-terminal truncated Q330X NEMO mutation. This patient developed early-onset PD with proteinopathies, including aggregations of αS, amyloid-*β*, tau, TDP-43, and ubiquitin (Furthmann et al., 2023). Wild-type NEMO, not the Q330X mutant, is recruited to αS aggregates and promotes M1-ubiquitination on itself and the aggregates. The Q330X truncation in NEMO decreases its association with HOIP and the ubiquitination of αS. In support of the above, KO of NEMO in murine embryonic fibroblasts (MEFs) promotes protein aggregate formation under proteasome/lysosome inhibition or heat stress (Furthmann et al., 2023).

### 6.5 Carboxyl terminus of Hsp70-interacting protein

CHIP is a U-box domain E3 (Jiang et al., 2001) with an N-terminal tandem tetratricopeptide repeat (TPR) domain that interacts with the HSP70 chaperone (Ballinger et al., 1999). CHIP has been found to colocalize with αS in LBs (Shin et al., 2005). In an H4 glioma cell model co-expressing aggregation prone truncated αS and synphilin-1 to induce αS aggregation (McLean et al., 2001), synphilin-1 interacts with αS to promote the formation of cytoplasmic inclusions resembling LBs found in PD. Using this model with proteasome or lysosomal inhibitors, CHIP was found to regulate αS turnover through two pathways: its TPR domain associates with HSC70 to promote proteasomal degradation, while its U-box domain facilitates lysosomal degradation. Notably, deletion of CHIP’s TPR domain leads to increased αS inclusion size (Shin et al., 2005).

In studies using BiFC assays to monitor αS oligomerization/aggregation, CHIP expression inhibits αS oligomerization and toxicity. Deletion of the TPR domain abolished these effects supporting Hsp70’s role in this process (Kalia et al., 2011; Tetzlaff et al., 2008). Additionally, CHIP can promote αS monoubiquitination in H4 cells and in cell-free systems incubated with the E2 enzymes UbcH5a/b (Kalia et al., 2011). BAG5 can negatively regulate this ubiquitination indirectly through its binding to Hsp70. Concomitant with the decreased ubiquitination, there is increased αS oligomerization. It remains unknown which αS Lys residues are ubiquitinated by CHIP.

### 6.6 Parkin

Parkin is an RBR-type E3 ubiquitin ligase extensively studied in PD as its mutation causes early-onset PD (Madsen et al., 2021). It is recruited to mitochondria upon activation of mitophagy and plays an important role in the process through its ubiquitination of mitochondrial membrane proteins. Its role in αS ubiquitination is controversial. Parkin has been identified in LBs and co-immunoprecipitation studies in human brain lysates revealed its association with UbcH7 and a novel 22-kDa O-linked glycosylated isoform of αS (αSp22), but not with the native 16-kDa αS monomer (Shimura et al., 2001). Although Parkin immunoprecipitated from normal brains was shown to promote αSp22 polyubiquitination, these findings have not been consistently reproduced by others (Chung et al., 2001) and so further investigation is warranted to clarify its role in αS ubiquitination.

### 6.7 E6-associated protein

E6-AP, a HECT E3, has also been found in LBs (Mulherkar et al., 2009). Its colocalization with αS, and ability to increase αS ubiquitination have been recapitulated *in-vitro*, using Neuro2a cells under proteasome inhibition (Mulherkar et al., 2009). In addition, E6-AP can increase the turnover of αS and reduce oligomer species in Cos-7 cells. Inhibitors suggest that this degradation occurs via the proteasome (Mulherkar et al., 2009). However, little is known about the type of ubiquitination stimulated by E6-AP, and its regulation *in vivo*.

### 6.8 UCH-L1 (PGP9.5)

UCH-L1 was first implicated in PD when a mutation was associated with familial disease (Leroy et al., 1998). However, recent studies challenge its monogenic role in PD, instead implicating UCH-L1 in spastic paraplegia, a neurodegenerative disorder characterized by optic atrophy and muscle weakness (Das Bhowmik et al., 2018; Rydning et al., 2017). Nevertheless, reduced levels of UCH-L1 have been reported in sporadic disease and the protein may play a role in regulating αS ubiquitination, suggesting its involvement in broader neurodegenerative processes.(Barrachina et al., 2006; Lowe et al., 1990; Yasuda et al., 2009). UCH-L1 co-immunoprecipitates with free and di-ubiquitinated αS species from rat brains (Liu et al., 2002). It can deubiquitinate αS derived from LB brain lysates (Sampathu et al., 2003) and its expression in cells promotes αS deubiquitination (Imai et al., 2000) and accumulation (Liu et al., 2002). Despite the documented DUB activity, it remains unclear whether UCH-L1’s actions modulate proteasome-mediated degradation of αS. UCH-L1 has been reported to interact with the CMA receptor LAMP2A and the I93M mutation enhances this association, thereby interfering with αS degradation (Kabuta et al., 2008). In a cell-free system, UCH-L1 has been reported to exhibit ubiquitin ligase activity, but this may be due to the high concentrations of enzyme and substrate used in the study (Liu et al., 2002).

### 6.9 Ubiquitin specific peptidase 8

The D442G activity enhancing mutation in USP8 is a cause of early-onset PD (Wu et al., 2023). This DUB can remove K6-linked ubiquitin chains from Parkin to activate it (Durcan et al., 2014) and can attenuate endosomal engulfment of membrane receptors by removing K63-linked ubiquitin chains (Mizuno et al., 2005). An additional role has been identified in HEK293T cells and iPSC-derived dopaminergic neurons where USP8 associates with αS primarily localized in early endosomes. Knocking down USP8 in SH-SY5Y cells promotes αS degradation through lysosomes and USP8 silencing in a Drosophila model of PD rescues αS-induced toxicity (rough eye phenotype, climbing function, and TH-positive neuron loss), while knockdown of the ESCRT I protein, Vps28, enhanced the rough eye phenotype (Alexopoulou et al., 2016). Most LBs stain with K63 but not K48 linkage specific anti-Ub antibodies (Alexopoulou et al., 2016). Interestingly, USP8 expression has been observed to be increased in the substantia nigra of PD patients, and the level of USP8-positive inclusions is negatively correlated with K63-Ub-positive inclusions (Alexopoulou et al., 2016). iPSC dopaminergic neurons derived from PD patients expressing this USP8 mutation exhibited higher overall levels of αS, with increased accumulation in the soma and decreased levels in dendritic regions (Wu et al., 2023). These phenotypes were exacerbated by overexpressing the USP8 hyperactive D442G mutant. D442G mutant knock-in models showed similar phenotypes, with homozygous mutants exhibiting stronger effects than heterozygous ones, confirming a dosage effect. Mechanistic studies revealed a stronger association between αS and the D442G mutant, leading to reduced K63-polyubiquitination of αS (Wu et al., 2023). These findings suggest an important role for K63-ubiquitination in mitigating αS neuronal toxicity in PD. Finally, the K63-specific DUB activity of USP8 was recapitulated *in vitro* using recombinant αS bearing K63 ubiquitin chains generated by NEDD4 (Alexopoulou et al., 2016). Taken together, these results suggest that USP8 interferes with endosomal degradation of αS by removing αS K63-linked ubiquitin chains.

### 6.10 Ubiquitin specific peptidase 13

USP13 expression has been observed to be increased in PD brains (Liu et al., 2019). Injection of mice with lentivirus expressing αS and USP13 or USP13 shRNA decreased or increased levels, respectively, of ubiquitinated αS detected by proximity ligation assay, compared to the non-injected hemisphere (Liu et al., 2019). Concomitant with the increased ubiquitinated αS, the USP13 KD also resulted in protection against dopaminergic neuronal death, and improved motor performance. Similar results were observed upon manipulating USP13 levels in the A53T αS transgenic mouse model of PD. The effects of KD in transgenic mice were mimicked by administration of a small molecule inhibitor of USP13 (Liu et al., 2022; Liu et al., 2021). Interestingly, the inhibitor increased proteasome activity in a dose-dependent manner in HEK cells expressing αS (Liu et al., 2022). These results suggest that USP13 can influence PD pathology by modulating αS ubiquitination and degradation. However, it remains unknown which type of ubiquitin linkage USP13 regulates and the downstream degradative pathway that is involved.

### 6.11 Ubiquitin specific peptidase 19

USP19 is a key modulator of an unconventional pathway of protein secretion that appears specific for misfolded proteins (MAPS). In this process, the ER localized isoform of USP19 deubiquitinates the misfolded protein and promotes its uptake into late endosomes in a process that involves Hsp70 and its co-chaperone DNAJC5/CSPα (Fontaine et al., 2016; Lee et al., 2023; Lee et al., 2016; Xu et al., 2018). Expressing αS or its disease-causing mutants in HEK293T cells results in secretion that can be stimulated by USP19 in a dose dependent manner and diminished by silencing of USP19. DNAJC5 associates with MAPS substrates, including αS, and facilitates their lysosomal degradation. Overexpression of dominant-negative (DN) ESCRT component (VPS4) abolished lysosomal degradation of αS, in support of DNAJC5 mediating αS degradation via endosomes. This DN VPS4 inhibition of lysosomal degradation enhanced MAPS secretion in the cells. Conversely, overexpression of DNAJC5 mutants associated with adult neuronal ceroid lipofuscinosis (ANCL) still facilitated αS endosomal/lysosomal degradation but failed to promote αS MAPS secretion (Lee et al., 2023). These results suggest that endosomal degradation and MAPS secretion represent alternative outcomes of cargo taken up by endosomes.

To explore these cellular observations *in vivo*, our group inactivated the USP19 gene in transgenic mice expressing the A53T disease causing mutation of αS (Schorova et al., 2023). Knockout of USP19 decreased accumulation of pS-129-αS aggregates, but increased ubiquitination of these aggregates as well as soluble forms of αS. This reduction in αS aggregate accumulation was also observed in USP19 KO primary neurons. Higher levels of αS oligomers were detected by proximity ligation assays in primary neurons and biochemically in the soluble fractions of KO brains. These results suggest that USP19 inhibits αS ubiquitination to facilitate αS aggregate formation. USP19 can cleave multiple types of polyubiquitin chain linkages – K6- (Liu et al., 2021), K11- (Jin et al., 2016; Tian et al., 2023), K27- (Lei et al., 2019), K48- (Harada et al., 2016; Tian et al., 2023), and K63-(Lei et al., 2019; Zhang et al., 2021), but the ubiquitin linkage type it targets on αS remains unclear.

## 7 Cell free studies of the effects of ubiquitination of αS on fibril formation

The establishment of *in vitro* fibrillization assays and the ability to generate ubiquitinated αS by synthetic chemical methods has permitted the exploration of the effects of these modifications on formation of αS fibrils in cell free systems. Hejjaoui et al. created monoubiquitinated αS at K6 by linking two protein fragments: one from *E. coli* (residues 19–140) and one synthetic (residues 1–18) using a specially modified lysine (*δ*-mercaptolysine) for ubiquitin conjugation (Hejjaoui et al., 2011). K6-monoubiquitinated αS significantly inhibited αS fibrillization *in vitro*, while its interactions with synthetic lipid vesicles or phosphorylation by its kinases CK1, GFK5, and PLK3 remained unaffected (Hejjaoui et al., 2011). K48-linked tetraubiquitin or diubiquitin chains conjugated at the αS K12 residue were similarly generated (Haj-Yahya et al., 2013). *In vitro* fibrillization assays showed that wild-type αS developed mature fibrils after extended incubation (8 days) as evaluated by TEM. In contrast, Tetra-Ub αS rapidly formed soluble but SDS-resistant aggregates within 48 h, which remained amorphous and unable to form amyloid fibrils after prolonged incubation, suggesting fibrillization blockade. In contrast to unmodified αS, di- or tetraubiquitinated αS at K12 could not be phosphorylated at S129 by PLK3, indicating disruption of the interaction with the kinase due to ubiquitination. Furthermore, both di- and tetra ubiquitinated αS were phosphorylated at Y125 by Syk, but this phosphorylation led to their precipitation, while similarly phosphorylated wild-type αS which remained soluble. Di- and tetraubiquitinated αS were efficiently degraded by the proteasome in HeLa cell extracts, whereas monoubiquitinated αS had its ubiquitin cleaved and was not degraded.

Meier et al. adopted a disulfide-directed ubiquitination approach, mutating individual αS lysine residues as well as the C-terminal glycine of ubiquitin to cysteine to permit linkage of ubiquitin to αS via disulfide bonds (Meier et al., 2012). Using this method, they generated monoubiquitinated αS variants (K6C, K10C, K12C, K21C, K23C, K32C, K34C, K43C, and K96C) for *in vitro* aggregation studies. Using thioflavin T fluorescence to monitor fibrillization, these studies revealed that while unmodified αS formed fibrils effectively, ubiquitination had varied effects depending on the modification site. K10C-Ub and K23C-Ub formed fibrils similar to wild-type αS, albeit with different kinetics. Modifications at K6C, K12C, and K21C inhibited fibril formation moderately, while ubiquitination at K32C, K34C, K43C, and K96C strongly inhibited fibril formation (Meier et al., 2012). Electron microscopy supported these findings, showing mature fibrils for unmodified, K10C-Ub, and K23C-Ub, while other sites formed short fibrils or amorphous aggregates. Notably, K96C-Ub formed oligomeric structures detected by the A11 anti-oligomeric antibody, indicating that ubiquitination at a specific site can inhibit fibril formation and promote oligomerization (Meier et al., 2012). Subsequent studies focusing on K6C-Ub, K23C-Ub, and K96C-Ub with more efficient and longer *in vitro* fibrillization protocols confirmed the inhibitory effects of these modifications on fibril formation, as evaluated by Thioflavin T (ThT) staining and TEM imaging (Moon et al., 2020). The stability of the fibrils was assessed by susceptibility to digestion by Proteinase K. This analysis confirmed the findings from ThT and TEM, suggesting that K23C-Ub promotes heterogeneous aggregate formation, while K96C-Ub results in a distinct fibril structure. Atomic force microscopy measurements showed that K96C-Ub fibrils had a shorter height compared to wild-type fibrils, further indicating structural differences (Moon et al., 2020). These findings collectively suggest that monoubiquitination of αS generally inhibits fibrillization and destabilizes protein aggregates.

## 8 Conclusions and future perspectives

This review has illustrated the significant progress made in our understanding of the roles of ubiquitination of αS (Tables 1, 2). A major function is its critical role in targeting αS to the endosomal/lysosomal system. The SCF-FBXL5 E3 complex and NEDD4-mediated K63-polyubiquitination can ubiquitinate fibrillar and endogenous αS, respectively, facilitating their uptake into endosomes and subsequent degradation in an NBR1 dependent manner. Consistent with this mechanism, αS colocalizes with endolysosomal vesicles, and its degradation is disrupted upon knockdown of key ESCRT machinery components, such as VPS28, VPS4, TSG101, CHMP3, and CHMP2B. Moreover, the deubiquitinase USP8, a counterpart to NEDD4, is also associated with endosomes and through its DUB activity inhibits *α*S lysosomal degradation. USP19/DNAJC5 promotes uptake of αS into late endosomes but this can promote secretion in addition to lysosomal degradation.

Although uptake of αS into endosomes plays a major role in targeting to lysosomes, macroautophagy still plays a role by clearing αS aggregates via aggrephagy. This involves LUBAC-mediated ubiquitination of αS, the aggrephagy adaptor proteins p62 and NBR1, and NEMO, all of which condense with linear ubiquitin chains at *α*S aggregates (Furthmann et al., 2023). Although there is abundant evidence for ubiquitination of αS targeting it to the lysosomal system, we cannot exclude a minor role for the proteasome in αS degradation, though such degradation is likely limited to non-oligomeric/aggregated forms of αS.

Beyond proteolytic degradation, several lines of evidence—albeit conflicting—suggest that ubiquitination may modulate αS aggregation. SIAH1/2-mediated mono-ubiquitination of αS can promote insoluble aggregate formation in αS overexpression cell models under proteasome inhibition (Lee et al., 2008; Rott et al., 2008). Conversely, USP19’s deubiquitination of αS can facilitate aggregate formation *in vivo* and in primary neurons with PFF seeding (Schorova et al., 2023). This USP19 regulation may be explained by increased deubiquitination of *α*S leading to its propagation through MAPS-mediated αS secretion (Lee et al., 2016) or direct structural effects of ubiquitin removal on αS (Schorova et al., 2023). Indeed, *in vitro* studies have shown that αS ubiquitination can disrupt the autonomous fibrillization process, not only slowing fibrillization but also leading to the formation of amorphous aggregates or oligomers.

In addition to the potential role of ubiquitination in *α*S aggregation, emerging evidence suggests that alterations in trafficking of the above mentioned αS containing lipid vesicles may contribute to its aggregation (Galvagnion et al., 2015; Kakuda et al., 2024; Stephens et al., 2023). Lipid components in LBs have long been recognized (Duffy and Tennyson, 1965; Forno and Norville, 1976; Gai et al., 2000), and more recent studies have emphasized the significance of lipid organelle aggregation, including autophagosomes, lysosomes, and mitochondria in LB formation (Mahul-Mellier et al., 2020; Shahmoradian et al., 2019). In addition, the prion hypothesis for the propagation of LB pathology indicates that the release and subsequent uptake of pathogenic forms of *α*S plays an important role in the progression of disease over time. This is supported by observations that propagation of disease in mouse models occurs most efficiently in the presence of both endogenous production of *α*S as well as exogenous delivery of pathogenic aggregates of αS (Luk et al., 2012; Luk et al., 2009). These two sources of αS would most likely first come in contact in endosomes and traffic together into lysosomes.

**Figure 4:**
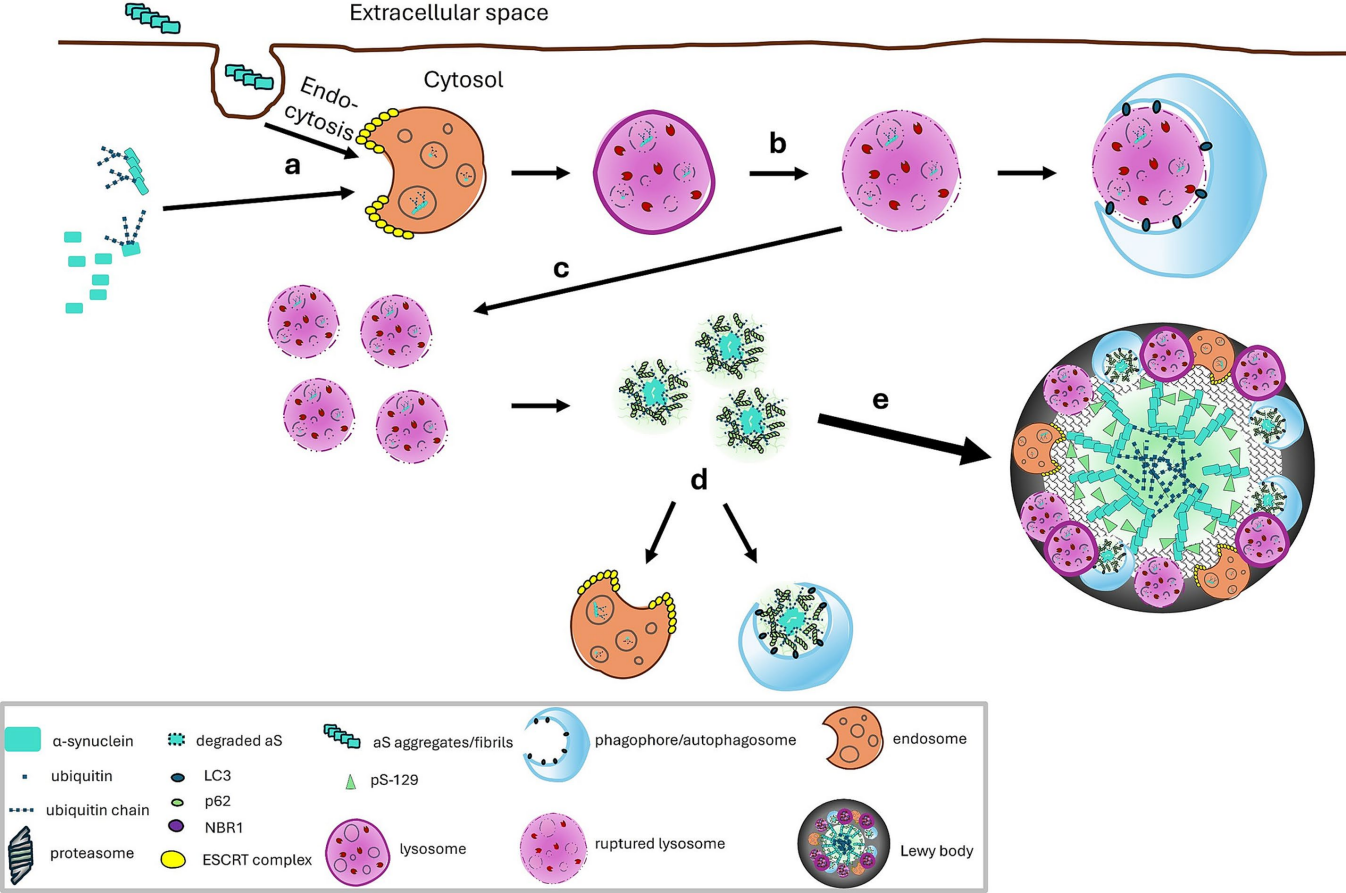
A proposed model for Lewy body formation: **(a)** Overloading of αS in endosomal vesicles can occur from overexpression of intracellular αS and uptake of extracellular αS. Ubiquitination of αS can facilitate the endosomal engulfment. **(b)** Rupture of endosomes or lysosomes in the cytosol is a stochastic event and in the absence of excessive loading in endosome, the released contents can be removed by macroautophagy (Kakuda et al., 2024) some of which depends on αS ubiquitination. Rupture occurs more frequently with overloading of αS and presence of pathogenic forms of αS (mutants, oligomers, aggregates). **(c)** With prolonged overloading of and more frequent rupture of endosomes, there is accumulation of fragments of endolysosomal lipid vesicles and creation of a low pH microenviroment which facilitates the seeding of αS (Buell et al., 2014; Flagmeier et al., 2016; Galvagnion et al., 2015; Grey et al., 2011; Kumari et al., 2021; Lee et al., 2005) into aggregate-prone species such as oligomers and truncated species. Autophagy adaptor proteins and free ubiquitin chains sequester aggregated αS, facilitate phase separation, providing additional seeds for aggregation. **(d)** Some of these species may become ubiquitinated which may impede fibrillization and promote degradation by aggrephagy or be retaken up by endosomes as in step (a). **(e)** With persistent overloading of the endosomes/lysosomes with these various forms of αS including pathogenic ones, a vicious cycle is created whereby aggregates continue to enlarge, particularly ones that have accumulated lipids and non-ubiquitinable proteins on the surface. Such surfaces lacking ubiquitination would prevent aggrephagy but lead to LBs which typically contain ubiquitinated αS located primarily in the core of the aggregate.

Integrating all these findings, we propose a model for LB formation (Figure 4). (a) Under normal, basal conditions, αS turnover may be mediated by multiple mechanisms including CMA and the ubiquitin dependent proteasome pathway. Any αS oligomers or aggregates formed because of the inherent propensity of αS to misfold may be cleared in lysosomes through ubiquitin dependent endosomal microautophagy and aggrephagic mechanisms. (b) Under abnormal conditions when there are increased levels of intracellular *α*S and its misfolded/oligomeric forms or increased uptake of extracellular αS, overloading of αS in endosomal vesicles occurs. (c) The high levels of αS in endosomes/lysosomes increases the risk of rupture of the vesicles with release of the contents including aggregation prone species (such as truncated αS generated potentially by lysosomal hydrolases, oligomers and fragmented fibrils) (Kakuda et al., 2024) and lipid fragments into the cytoplasm, creating a low pH microenvironment. This microenvironment facilitates the seeding of *α*S (Buell et al., 2014; Flagmeier et al., 2016; Galvagnion et al., 2015; Grey et al., 2011; Kumari et al., 2021; Lee et al., 2005) into aggregate-prone species. This in turn recruits autophagy adaptor proteins and free ubiquitin chains which sequester the aggregated *α*S, facilitate phase separation and provide additional seeds for aggregation. (d) Some of these species may become ubiquitinated which may impede fibrillization and promote degradation by aggrephagy or be retaken up by endosomes as in step (a). (e) With persistent overloading of the endosomes/lysosomes with these various forms of *α*S including pathogenic ones, a vicious cycle is created whereby aggregates continue to enlarge, particularly ones that have accumulated lipids and non-ubiquitinable proteins on the surface which would prevent aggrephagy but lead to LBs which bear ubiquitinated *α*S located primarily in the core of the aggregate.

To explore this model and the mechanisms involved, cellular systems which can be maintained for prolonged periods to allow LB like aggregates (e.g., positive staining for pS129-*α*S, ThT, or the association of membranous organelles with the concentric protein structure) to form are required. At this time, the best systems are primary neurons exposed to αS preformed fibrils and iPSC-derived neurons from PD patients bearing *α*S mutations. These neurons can spontaneously form pSyn aggregates and induce mitochondrial and synaptic dysfunctions (Diao et al., 2021; Lin et al., 2016; Ludtmann et al., 2018). Furthermore, Tanudjojo et al. demonstrated that iPSC-derived neurons can effectively form LB-like aggregates upon extracellular PFF seeding (Tanudjojo et al., 2021), The ability to apply to these systems genetic tools or specific inhibitors/activators that target specific steps in these pathways will help identify critical steps in the pathogenesis of toxic forms of *α*S and LBs. These could ultimately lead to development of therapies that could halt the progression of PD at early stages of disease when there is minimal functional impairment.

The above model seeks to explore comprehensively the role of αS ubiquitination in LB formation. It should be noted though that other potential effects of this ubiquitination remain unexplored. For example, it is unclear whether αS ubiquitination affects the physiological functions of αS monomers and tetramers, impacts intracellular trafficking (e.g., between synapses and neuronal soma) and secretion of pathological aggregates, or interacts with other post-translational modifications. Finally, there is ongoing debate regarding whether LBs are neurotoxic or represent a protective mechanism that sequesters toxic oligomers and aggregates.
